# The feasibility and effectiveness of a blended-learning course for detecting and avoiding bias in medical data: a pilot study

**DOI:** 10.1186/s12909-020-02332-w

**Published:** 2020-11-07

**Authors:** Cora Koch, Jochen Brich

**Affiliations:** Clinic of Neurology and Neurophysiology, Medical Center – University of Freiburg, Faculty of Medicine, University of Freiburg, Breisacher Str. 64, 79106 Freiburg, Germany

**Keywords:** Conflict of interest, Bias detection, Scholarship, Blended learning, Key feature test, Medical education

## Abstract

**Background:**

Conflicts of interest (COIs), including those arising from interactions with pharmaceutical companies, may lead to bias in medical data. Although medical students are now requesting more education on COIs and bias, they are still not adequately taught during medical school, and few published courses on this topic exist*.* The objective of our study was therefore to evaluate the feasibility and effectiveness of a blended-learning course for detecting and avoiding bias in medical data, with a special focus on COIs.

**Methods:**

We developed a blended learning course on bias detection, COIs, and risk communication. It was piloted in the Fall Semester of 2019/2020 using a pre/post-test design. The primary outcome was a gain in bias detection skills, tested by a novel key feature test. Secondary outcomes were (i) skepticism (tested using an attitude questionnaire), (ii) the intention to manage COIs in a professional way so as to avoid bias (tested using a situational judgment test) and (iii) the course evaluation by the students.

**Results:**

Seventeen students participated in the study. The key feature test showed a significant improvement in bias detection skills at post-testing, with a difference in means of 3.1 points (95%-CI: 1.7–4.4, *p*-value: < 0.001; highest possible score: 16 points). The mean score after the course was 6.21 (SD: 2.62). The attitude questionnaire and situational judgment test also showed an improvement in skepticism and intentions to manage COIs, respectively. Students evaluated the course as having been worthwhile (Median: 5, IQR: 0.75, Likert-Scale 1–6, 6 = fully applicable).

**Conclusions:**

The blended learning format of the course was feasible and effective. The results suggest a relevant learning gain; however, the low mean score on the key feature test after the course reflects the difficulty of the subject matter. Although a single course has the potential to induce significant short-term improvements in bias detection skills, the complexity of this important subject necessitates its longitudinal integration into medical curricula. This concept should include specific courses such as that presented here as well as an integration of the topic into clinical courses to improve context-related understanding of COIs and medical data bias.

**Supplementary Information:**

The online version contains supplementary material available at 10.1186/s12909-020-02332-w.

## Background

Conflicts of interest (COIs) are a possible cause of bias in medical data. Although they can arise from different issues, COIs resulting from interactions with pharmaceutical companies occur frequently and their effects are well-studied. Indeed, there is a large body of evidence to suggest that such interactions carry a risk for bias with regard to clinical trial results, interpretation of data, and clinical decision-making, thus impacting all aspects of medical practice [1–6]. In addition, several studies have shown that contact between the medical profession and pharmaceutical companies begins early, with medical students already reporting that they have interacted at some point with pharmaceutical companies, suggesting that COIs should be part of the medical school curriculum [7, 8].

Medical students have now become more vocal about the need to regulate such COIs, as well as for education on how to manage them. According to surveys conducted by medical student associations, universities in the US have started to introduce some courses on this subject; however, they are still lacking at most German universities [9–11].

At the same time, German regulatory bodies have emphasized the need for more education on scholarship in medicine, which includes, among other aspects, competencies and attitudes necessary for understanding, assessing and applying scientific methods and data [12]. “Scholar” is also one of the seven roles in the CanMEDS Framework, referring to “teaching others, evaluating evidence and contributing to scholarship”. It was revised in the 2015 Framework to place more emphasis on the competency of critical appraisal of evidence [13]. Considering the relevance that COIs have on the generation and presentation of scientific data, courses focused on competencies related to scholarship should include content that is related to COIs.

Nonetheless, evidence-based guidance on how to design such courses is limited [14]. Evaluation of one particular curriculum in a German randomized controlled trial found that the combination of COI education and shared decision-making led to significant learning gains in terms of risk communication competency [15]. However, this curriculum was designed as a block course, which is not feasible for every medical school. Blended learning, defined as “the thoughtful integration of classroom face-to-face learning experiences with online learning experiences” by Garrison and Kanuka (2004), offers more flexibility for integration into the curriculum due to the reduction of time needed for face-to-face interaction with teaching personnel [16]. Compared to face-to-face instruction only, blended learning especially offers advantages for learners with heterogeneous prior knowledge, as is the case for COIs and bias detection in our curriculum and likely in other curricula, too. The online preparation ensures students arrive to the face-to-face session with a similar knowledge base. This allows for a more productive session that can be used for interactive activities such as case-based learning and discussions, which are especially important for teaching controversial subjects such as management of COIs.

Our objective was therefore to design a blended learning course for teaching students to detect and avoid bias in medical data, with special emphasis on COIs that specifically arise from interactions with the pharmaceutical industry. A pilot study was conducted to evaluate the feasibility and effectiveness of this course.

## Methods

### Ethics

The study was approved by the local Ethics Committee of the University of Freiburg (Application number: 326/19).

### Curricular context

Medical school in Germany is structured into a pre-clinical and clinical phase lasting two and 4 years, respectively, with the last year of the clinical phase consisting solely of clinical clerkships. German medical schools differ widely as to the structure and timing of teaching subjects related to scholarship. At the University of Freiburg, students take the course “Thinking and Acting Scientifically” in their first year and a course on medical statistics and epidemiology in their 5th year of study. Medical students can choose to pursue a doctorate degree by completing a doctoral thesis during or after the completion of medical school but are not required to do so. In a survey from 2017 among medical students, 57% (1302/2291) indicated that they are currently working on their doctorate thesis or have completed it, while 66% (653/998) of the remaining students indicated that they would like to pursue a doctorate degree in the future [17].

### Study design

A single-center, uncontrolled pilot study with a pre/post-test design was performed. The course was offered on a voluntary basis and independently of other courses. Immediately after completing the course, students participated in a summative test and then evaluated the course using a standard evaluation questionnaire. The primary outcome was the score on a key feature test that was developed to assess the students’ competency in detecting bias in medical data. The secondary outcomes were the results of (i) a situational judgment test (SJT), (ii) an attitude questionnaire, and (iii) the course evaluation by the students. At pre-testing, the questionnaires also included sociodemographic items as well as questions regarding whether the students had begun or were planning on doing a doctorate thesis.

### Recruitment

Participants were recruited during the medical course lectures for 4th year students, as well as via online information on the university’s learning management system “ILIAS”. Although 4th-year medical students were the main target group, students in the 5th or 6th years of the course were also allowed to enroll. Students had to be currently enrolled at the University of Freiburg. Medical students in their 3rd year or below were excluded. All students meeting these inclusion criteria who gave consent to participate were included in the study.

### Course development

Course development was based on the 6-step approach described by Kern et al. [18]. The curriculum was modeled on one that was previously co-developed by the current first author (CK), which was found to be effective in a randomized controlled trial [15] and subsequently adapted into a blended learning format for the present study. Due to time constraints, but also to ensure that the course remained concise, some statistical concepts, most of which were related to screening (i.e. sensitivity/specificity), were omitted in favor of those related to therapeutic trials. In addition, more emphasis was placed on the management aspect of COIs.

The resulting course consisted of 6 units, four of which included one 30-min online module and one 1.5-h face-to-face session. The first and last units each consisted of a 45-min face-to-face session. The units were divided into three sections. The section “Why?” covered the question of why data might be biased and how COIs should be managed to prevent them causing bias. The section “How?” covered how specific aspects of study design and the presentation of statistics might lead to biased interpretation of the data. The last section, “Transfer into Clinical Practice”, asked students to apply their knowledge by advising a patient on a therapeutic choice in an unbiased way. Didactically, the online modules consisted of interactive pdf-documents that included individual knowledge assessments, while the face-to-face sessions employed a variety of didactic instruments such as group discussions, analysis of scientific publications, and role play (see Table 1 for a short overview and Additional file 1 for a detailed overview of the course content and teaching/learning activities).

**Table 1:** Overview of the main course-learning goals and corresponding teaching/learning activities and assessment

Major learning goals	Teaching/learning activities	Assessment
Students aim to manage conflicts of interest so as to avoid bias.	Students discuss how they would manage conflicts of interest in a setting of their choice.	Attitude questionnaire and situational judgment test
Students are able to detect bias in a study design and in data presentation.	Students read clinical trial publications as well as advertising material and present the biases they find to classmates.	Key Feature Test
Students apply their knowledge of bias by choosing to discuss relevant, balanced data in consultations with patients.	Students design a fact box and carry out a mock consultation based on biased informative material.	Peer assessment of a mock consultation

### Assessment design

According to the theory of constructive alignment, assessment should be tailored to the learning goals that the students are expected to achieve [19]. We therefore used three different types of assessment at the end of the course (see Table 1). An attitude questionnaire and a SJT were applied to assess whether students had acquired professional attitudes and intentions to manage COIs, while a key feature test was developed and implemented (see below) to assess whether students were able to detect biases in data or study designs. The transfer into practice was assessed with structured peer feedback after the mock consultation. The attitude questionnaire, SJT and key feature test were also used at pre-testing to allow for a pre−/post-comparison.

### Development of the key feature test

The key feature test served as the primary outcome because it assessed the most important learning goal of the course, namely, the detection of bias in data or study design. We opted to use a key feature test to assess bias detection because we wanted to assess the application of the competency in clinically relevant scenarios rather than simply assessing knowledge. A key feature test offers an objective, reliable way to assess this competency while focusing on important and difficult “key features”, i.e. critical steps in a decision-making process where the most errors are made [20, 21]. In the case of bias detection, this allows for a focus on frequent and relevant forms of bias that are often overlooked. Our key feature test was loosely based on the guidelines by Page and Bordage [20]. Because key feature tests were originally designed to test clinical decision-making skills in medical students, some adjustments had to be made. The key feature problems were originally meant to allow for testing a broader range of clinical cases by focusing on the critical steps of each case. Bias, however, is not specific to a certain illness or clinical scenario. The same type of bias, such as outcome reporting bias, can occur in different scenarios (i.e. when a pharmaceutical representative presents information in a brochure or in a scientific publication). For our purposes, we first developed a two-dimensional blueprint: one dimension consisted of the category from which bias in data can result (study design, data presentation and graphics), along with the key learning goals (~key features) that belong in each of these categories, while the second dimension consisted of the scenarios in which such bias becomes relevant to clinical practice (such as in a conversation with a pharmaceutical representative or when researching a medication requested by a patient). The number of questions for each category was based on the corresponding number of learning goals.

The final key feature test consisted of 16 questions based on five cases. Seven of these were “long-menu” questions, and nine were “short-menu” questions. See Additional file 2 for an example.

### Secondary outcomes

#### Attitude questionnaire and skepticism score

The attitude questionnaire was a 10-item questionnaire previously adapted from Sierles et al. to the situation in Germany [8, 22]. The adaptation of the questionnaire consisted in a translation into German and adjustment of certain terms that are not applicable for the situation in Germany. The skepticism score was calculated from a selection of six of the attitude items, in accordance with the method by Sierles et al. The score ranges between 0 and 1, where higher values indicate higher skepticism [22].

#### Situational judgment test

The SJT was used to assess intentions regarding the professional management of COIs that result from interactions with the pharmaceutical industry. It was previously developed to assess a different curriculum [15], and encompasses five scenarios, each describing a COI and five possible ways to behave in each. Students were asked to rate each behavioral option on a Likert scale from 1 to 6, according to how likely they thought it was that they would behave in that way in the given situation. The most desirable behavioral option was determined by expert consensus, and students gained points depending on how they rated the likelihood of behaving in a more vs. less desirable way. A maximum score of 125 points was possible, where higher scores indicated better intentions for managing the COI. See Additional file 3 for an example of one of the SJT scenarios.

#### Evaluation

Students were asked to evaluate the course anonymously using an adapted standard questionnaire (based on the Trierer Inventar zur Lehrevaluation/Trier Inventory for Evaluation of Teaching) [23]. The original questionnaire was adapted by deleting not only the items that were not relevant to the present course, but also sociodemographic items that had been addressed previously. The adapted version consists of 29 items evaluated on a Likert scale, and two open-ended questions. Twenty-four of the Likert scale items are divided into five dimensions (structure and didactics, impulse and motivation, interaction and communication, personal benefits from the course, and practical relevance), while the remaining five items are not attributed to any particular dimension, including one item which asks for a global assessment of the course. In addition, students were asked to provide informal verbal feedback at the end of the course.

### Statistical analysis

Data was included for analysis if it arose from students who had completed the key feature test both before and after the course. The pre- and post-test scores from the key feature test, skepticism test and SJT were each compared using paired 2-tailed t-tests. Descriptive results are reported for the individual attitude questionnaire items. Cronbach’s alpha was calculated to assess the internal consistency of the key feature test. The discriminatory power of single items was assessed using the corrected Pearson-Brevais correlation coefficient. These statistics were recalculated after removing items with a negative discriminatory power in two iterations until all remaining items had a positive discriminatory power. Mean item scores were calculated to assess item difficulty. Descriptive results are provided for the Likert scale items, which were analyzed according to dimensions. The results of the open-ended questions are reported qualitatively. Statistical analysis was performed using the R Environment for Statistical Computing, Version 3.6.2 [24].

## Results

### Participants

Seventeen students participated in the study. The average age was 26.4 years (SD: 3.69), with nine (52.9%) females and eight (47.1%) males. The median semester of study was the eighth (IQR 1, *n* = 16). The majority of students (15/17, 88.2%) had either begun (6/17, 35.3%) or were planning on doing (9/17, 52.9%) a doctorate thesis. Of the 26 students who initially showed interest in the course, three could not participate due to schedule conflicts, one was unaware that the course was offered as part of a study and thus declined to participate, and five did not provide any reasons for their decision not to participate.

### Primary outcome – key feature test

#### Item analysis

The original key feature test had a Cronbach’s alpha of 0.592. The discriminatory power of items ranged between − 0.23 and 0.56, with nine items having a good discriminatory power of > 0.2. One item was answered incorrectly by all of the students, meaning its discriminatory power could not be calculated. Removing items with a negative discriminatory power resulted in a Cronbach’s alpha of 0.75, and all remaining items had a discriminatory power of > 0.2. Of the five items for which the discriminatory power was negative or could not be calculated, two contained an error and three contained ambiguous wording. The original level of item difficulty ranged between 0 and 0.76, with eight items carrying an acceptable level of difficulty between 0.4 and 0.8. The difficulty ranged between 0.12 and 0.76 for the 11 items with a positive discriminatory power, where seven had an acceptable level of difficulty.

#### Results

**Fig. 1:**
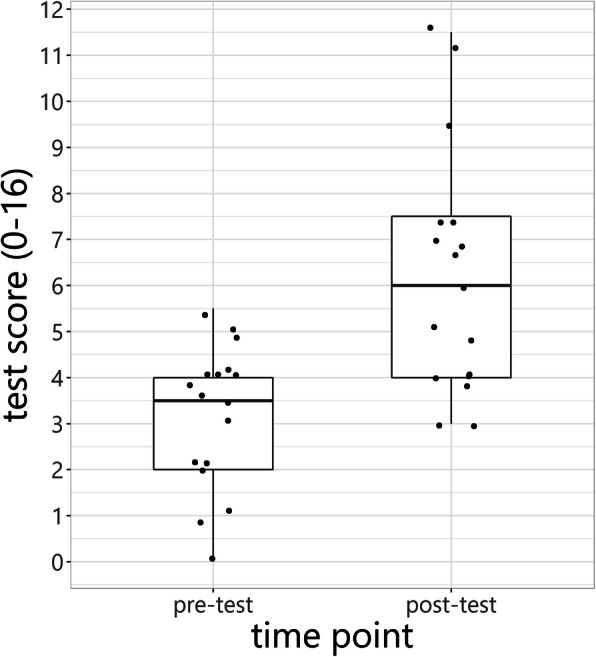
Box plot of results of the key feature test

The results of the key feature test were significantly better after the course (mean: 6.21 (SD: 2.62)) than before the course (mean: 3.15 (SD: 1.57)), with a difference in means of 3.1 points (95%-CI: 1.7–4.4, *p*-value: < 0.001) (Fig. 1). A sensitivity analysis that only used items with a positive discriminatory power revealed a difference in means of 2.4 (95%-CI: 1.2–3.6, *p*-value: 0.001), and mean scores of 2.3/11 (SD: 2.5) and 4.7/11 (SD 1.3) before and after the course, respectively.

### Secondary outcomes – attitudes

The skepticism score was higher after the course, with a difference in means of 0.12 (95%-CI: 0.077–0.17, *n* = 14); this indicated that following the course, students had become more skeptical of interactions with pharmaceutical companies. The mean skepticism score was 0.48 and 0.61 at pre- and post-testing, respectively. Additional Figure 1 and Additional Table 2 present the students’ answers to individual items at the pre- and post-test time points (see Additional files 4 and 5).

### Secondary outcomes – situational judgment test

After the course, the students had a higher SJT score (mean: 74.7 (SD: 15.6)) than that before the course (mean: 64.3 (SD: 11.0)), with a difference in means of 10 points (95%-CI: 3.9–17, *n* = 17).

#### Evaluation

Table 2 gives an overview of the quantitative evaluation by the students according to the domain studied. There were four further items that were not part of any domain. For the global assessment, students evaluated the course as having been worthwhile, with a median score of 5.0 (IQR 0.75) (6 = fully applicable, 1 = not applicable). For the item on whether the students regularly prepared for the class, the median answer was 5 (IQR: 1) (same scale). For the item on whether the students regularly followed-up on the class by individual reading, the median answer was 2 (IQR: 2) (same scale). Regarding the availability of the teacher for questions outside of the class, the median answer was 6 (IQR: 1) (same scale). Students generally evaluated the course requirements as being adequate, with a median score of 3.0 (IQR: 0) on a scale from 1 (too easy) to 6 (too difficult).

**Table 2:** Quantitative results of the student evaluations for different domains

Domain	median	IQR
Structure and didactics	5.0	1.00
Impulse and motivation	6.0	1.00
Interaction and communication	6.0	1.00
Personal benefits of the course	5.5	1.00
Practical relevance	6.0	1.00

Individual questions for each domain were analyzed together. Higher scores indicate a better evaluation, Likert scale from 1 to 6

In their answers to the open-ended questions, 5/17 (29.4%) students mentioned the high practical relevance of the course and/or the importance of the topic, and 5/17 (29.4%) commented positively on the online modules. Six of 17 (35.3%) students said that they would have preferred to spend more time on statistics, while 3/17 (17.6%) remarked that there didn’t appear to be enough time to cover all topics. The discussions in the course were controversial, with two students (11.8%) commenting that they enjoyed them, one (5.9%) commenting that there were too few of them, and one (5.9%) commenting that there were too many. Additional comments mostly pertained to other aspects of didactics such as course structure or the use of mock consultations (seven positive and three negative comments).

## Discussion

This pilot study demonstrates that a blended-learning course on detecting and avoiding bias in medical data with a focus on conflicts of interest is feasible and leads to significant learning gains regarding the detection of bias in medical data as well as affecting attitudes and intentions related to the professional management of COIs. The course generally received a positive evaluation from the students, with all aspects rated with a median score of at least 5/6. However, the low mean post-course score on the key feature test highlighted the difficulty of the subject matter.

We believe that the combination of difficult subject matter and the chosen test format were the two main reasons for the low post-course scores. A lack of motivation on the students’ part appears to be less likely, since the course was optional with no course credits available, and the only incentive was the chance to win a book voucher, which instead is suggestive of a high intrinsic interest in the subject matter. In addition, students indicated on the evaluation that they usually prepared for the course using the e-modules. The positive evaluation by the students further suggests that the course was well-crafted, making it unlikely that the low post-course score was due to inadequate design.

In principle, the medical students in Freiburg should have been optimally prepared for the course through a class named “Thinking and Acting Scientifically”, which they are required to take in their second semester of study [25]. In this class, students learn to explain basic methodological aspects of medical trials, define and interpret basic statistical parameters, and read and critically assess publications. In our course, some of these concepts were intentionally repeated, but others were assumed to have already been mastered by the students. However, it became clear during the course that students were still overwhelmed by some tasks, such as the interpretation of basic statistical concepts or the analysis of a published clinical trial. In our opinion, this underscores the need for longitudinally integrating such subject matter into the medical curriculum, with the repetition of specific classes on the topic and the integration of medical data bias and COIs into classes in a clinical context.

The design of the key feature test in the present study may also be part of the reason for the low post-course scores. It has been hypothesized that key feature problems are capable of testing higher-level cognitive processes than more common tests such as multiple-choice exams [26, 27]. In at least one other study, this may have been one reason why students received a lower key feature test score than that obtained in a multiple-choice exam on the same subject [28].

It is difficult to compare the results of the key feature test to other studies because according to our literature search results, a key feature test has never been used to test for the detection of bias in medical data. There are essentially no publications available that describe tests for this specific type of competency, underscoring the novelty and importance of our results. However, studies on blended learning courses for teaching other topics did find a comparable effect size [29]. Regarding the secondary outcomes of attitudes and the SJT, the changes were similar to those reported for a previously published curriculum, albeit slightly smaller: students became more skeptical of interactions with pharmaceutical companies and their intentions to manage COIs in a professional manner improved [15]. Considering that the previous curriculum was a course comprising 19 h, and the current course encompassed only 10 h (including preparation time), this is a remarkably good outcome.

A strength of the present study was the use of pre- and post-tests to objectively evaluate effectiveness. However, there were also several limitations. Due to the pilot nature of the study, there was no control group; therefore, we could not control for confounding or intervening variables and the improvement in the key feature test scores may have been partly due to a learning effect arising from taking the same test twice. However, due to the complex nature of the test, the lack of feedback after the pre-test, and the fact that the questions were not published, we assume that this effect was minimal. In addition, the sample size was small, thus it is unclear whether the effects found in this group can be extrapolated to other groups. Finally, the newly developed key feature test has not been validated, although the test statistics for reliability were satisfactory.

For future versions of the course, the difficulty of the subject matter will be accounted for by building up to complex tasks in a more gradual way, beginning with exercises that repeat previously learned material in more depth. In addition, we plan to extend the time spent on statistics, since the general lack of statistical understanding seemed to be a major hinderance during the course, and also because students indicated in the evaluation that they would prefer more instruction on statistics. Finally, the key feature test will be improved by editing several questions with low discriminatory power to improve unclear wording or other errors. It also needs to be determined whether an increase in the number of questions leads to better reliability (a Cronbach’s alpha of > 0.8 would be optimal), although simply improving the existing questions may be enough to achieve this goal [30]. In the future, the course will be offered as an elective course for doctoral candidates, so that it will reach more students.

## Conclusions

A blended-learning course is a feasible and effective way to teach students how to detect and avoid bias in medical data. However, even though the participating students should have been well prepared for the course due to previous instruction in the subject matter, they still produced low scores in the post-test. In our view, this underscores the need for longitudinal integration of the subject into medical school curricula; courses targeting specific competencies related to scholarship in medicine at only one or two points in the curriculum will not be sufficient. Instead, it is necessary to additionally integrate the subject matter into the clinical courses. In a future study, we intend to reassess an adjusted version of the course using a more rigorous design with more participants.

## Supplementary Information

**Additional file 1: Table S1.** Detailed overview of the course

**Additional file 2.** Example of a key feature case

**Additional file 3.** Example of a situational judgment test

**Additional file 4: Figure S1.** Individual item results of the attitude questionnaire

**Additional file 5: Table S2.** Individual item data for the attitude questionnaire.

## Data Availability

The datasets used and analyzed during the current study are available from the corresponding author upon reasonable request.

## Abbreviations

COI: Conflict of interest
SJT: Situational judgment test
