# Supplementary material for: The feasibility and effectiveness of a blended-learning course for detecting and avoiding bias in medical data: a pilot study
Source: BMC Med Educ. 2020 Nov 7;20:408. doi: 10.1186/s12909-020-02332-w (PMC7648418; doi:10.1186/s12909-020-02332-w)
Supplement: Supplementary file 1 — Additional file 1: Table S1. Detailed overview of the course [file 12909_2020_2332_MOESM1_ESM.docx]

Additional Table 1: Detailed overview of the course

| **Topic** | **Teaching/Learning Activity** | **Time (min)** |
| --- | --- | --- |
| **Section 1: Why?** |  |  |
| *Unit 1: Introduction* |  |  |
| Conflicts of interest in medicine: definition, frequency, effects | Presentation | 45 |
| *Unit 2: Psychological mechanisms related to conflicts of interest* |  |  |
| Definition of cognitive bias, especially self-serving bias Psychology of influence: reciprocity, consistency, social desirability, sympathy, and authority | Online-Module | 30 |
| Online-Module Recap | Pairwise discussion of case vignettes | 15 |
| Management of conflicts of interest to prevent bias | Group discussion relating to a specific context in which conflicts of interest may lead to bias | 105 |
| **Section 2: How?** |  |  |
| *Unit 3: Bias in Study design* |  |  |
| Review of important aspects of randomized controlled trials: control group, endpoints and surrogate endpoints, power analysis | Online-Module | 30 |
| Online-Module Recap | Multiple choice questions answered in a pairwise manner and discussed within the group | 5 |
| Frequent weaknesses in study design that may lead to biased results | Presentation | 25 |
| Detection of study design elements that may lead to biased results | Group work (analysis of a published randomized controlled trial) | 60 |
| *Unit 4: Bias in data presentation* |  |  |
| Review of commonly used statistics to report trial results: relative and absolute risk reduction, p-value, confidence intervals, standardized mean difference | Online-Module | 30 |
| Intention-to-treat analysis |  |  |
| Online-Module Recap | Calculation exercises solved individually and discussed within the group | 5 |
| Frequent biases in data presentation | Presentation | 25 |
| Detection of biased data presentation in pharmaceutical company information | Group game | 60 |
| **Section 3: Transfer into clinical practice** |  |  |
| *Unit 5: Risk communication* |  |  |
| Risk communication - definition and best practice | Online-Module | 30 |
| Online-Module Recap | Bringing the elements of risk communication into the correct order (pairwise) | 5 |
| Fact boxes | Presentation, group work | 40 |
| Risk communication - practical application | Mock consultation | 45 |
| *Unit 6: Risk communication* |  |  |
| Risk communication- practical application | Mock consultation based on biased case descriptions | 45 |
