# Supplementary material for: The feasibility and effectiveness of a blended-learning course for detecting and avoiding bias in medical data: a pilot study
Source: BMC Med Educ. 2020 Nov 7;20:408. doi: 10.1186/s12909-020-02332-w (PMC7648418; doi:10.1186/s12909-020-02332-w)
Supplement: Supplementary file 2 — Additional file 2. Example of a key feature case [file 12909_2020_2332_MOESM2_ESM.docx]

**Example of a key feature case**

You are treating a 64-year-old patient, Mr. Mueller, on the stroke unit. He has suffered a microangiopathic stroke and is at high general cardiovascular risk (arterial hypertension, type 2 diabetes, obesity, hypercholesterolemia). As there is no evidence of a cardioembolic stroke, Mr. Mueller is receiving aspirin for secondary prevention of further strokes. Normally you would also recommend “normostatin” (a cholesterol-lowering drug) because it has been shown to lower the risk of recurrent strokes. However, Mr. Mueller is skeptical because he has a friend who reacted to “normostatin” with muscle pain. He therefore wants a different medication. “Niedrestin”, which was introduced to you by a pharmaceutical representative, was recently approved for marketing. In a four-armed approval study, niedrestin was compared to normostatin in three different doses (randomization 1:1:1:1, n= 5.024). The medium and high doses led to considerably lower levels of cholesterol than those associated with normostatin treatment (see figure from the pharmaceutical brochure). The figure contains the results of the intention-to-treat analysis.

1. What type of endpoint is the cholesterol level? (long-menu question, correct answer: surrogate endpoint)
2. In your opinion, the results of which endpoint would be the most relevant for judging how effective niedrestin is in comparison to “normostatin”?
   1. Cholesterol level
   2. Patient's weight
   3. Thickness of cholesterol deposits in the cervical arteries
   4. Incidence of strokes
   5. Incidence of muscle pain or related complaints
   6. Blood sugar
   7. Blood pressure
3. The following table shows the frequency of muscle pain that was detected during the study. The pharmaceutical representative uses this table to demonstrate that muscle pain is less common with niedrestin use. Which aspect of the study design presumably leads to bias in the data with regard to this aspect?

| **Medication and dosage** | **Muscle pain % (n)** |
| --- | --- |
| Niedrestin, 10 mg | 2% (25/1256) |
| Niedrestin, 20 mg | 4% (50/1256) |
| Niedrestin, 40 mg | 6% (75/1256) |
| **Niedrestin, total** | **4% (150/3768)** |
| **Normostatin, 40 mg** | **5% (63/1256)** |

- 1. Outcome selection
  2. Inadequate sample size
  3. Lack of confidence intervals
  4. Low power
  5. Dosage of control therapy
  6. Wrong research question
  7. Wrong protocol for statistical analysis
