# Supplementary material for: The feasibility and effectiveness of a blended-learning course for detecting and avoiding bias in medical data: a pilot study
Source: BMC Med Educ. 2020 Nov 7;20:408. doi: 10.1186/s12909-020-02332-w (PMC7648418; doi:10.1186/s12909-020-02332-w)
Supplement: Supplementary file 3 — Additional file 3. Example of a situational judgment test [file 12909_2020_2332_MOESM3_ESM.docx]

**Example of a situational judgment test**

Following, different scenarios that you may be confronted with during your medical education or as a medical practitioner are first described. Thereafter, different options for how you might react will be presented. Please judge how you would behave for each option. Note that you are judging each option separately. Your answers may contradict each other.

You are doing a rotation on a general internal medicine ward. The resident who is supervising you is invited to a meal by a pharmaceutical representative. She asks the representative whether you as a future doctor may also come along and they invite you to dinner as well. How would you most likely behave?

|  | Never | Highly unlikely | Unlikely | Likely | Highly likely | Definitely |
| --- | --- | --- | --- | --- | --- | --- |
| I would accept the invitation because I could then find out about new medications from the pharmaceutical representative and learn a lot in general. | □ | □ | □ | □ | □ | □ |
| I would accept the invitation because the representative's views would have little influence on me, since I am not yet allowed to prescribe medication | □ | □ | □ | □ | □ | □ |
| I would not accept the invitation because I would want to remain independent. A meal with a pharmaceutical representative could subconsciously influence me. | □ | □ | □ | □ | □ | □ |
| I would accept the invitation because I am already aware that the pharmaceutical representative is trying to influence me as a future doctor, hence I would be immune to this influence. | □ | □ | □ | □ | □ | □ |
| I would accept the invitation to reward myself for the extreme effort I put into my studies. | □ | □ | □ | □ | □ | □ |
