# Supplementary figures and images for: The feasibility and effectiveness of a blended-learning course for detecting and avoiding bias in medical data: a pilot study
Source: BMC Med Educ. 2020 Nov 7;20:408. doi: 10.1186/s12909-020-02332-w (PMC7648418; doi:10.1186/s12909-020-02332-w)

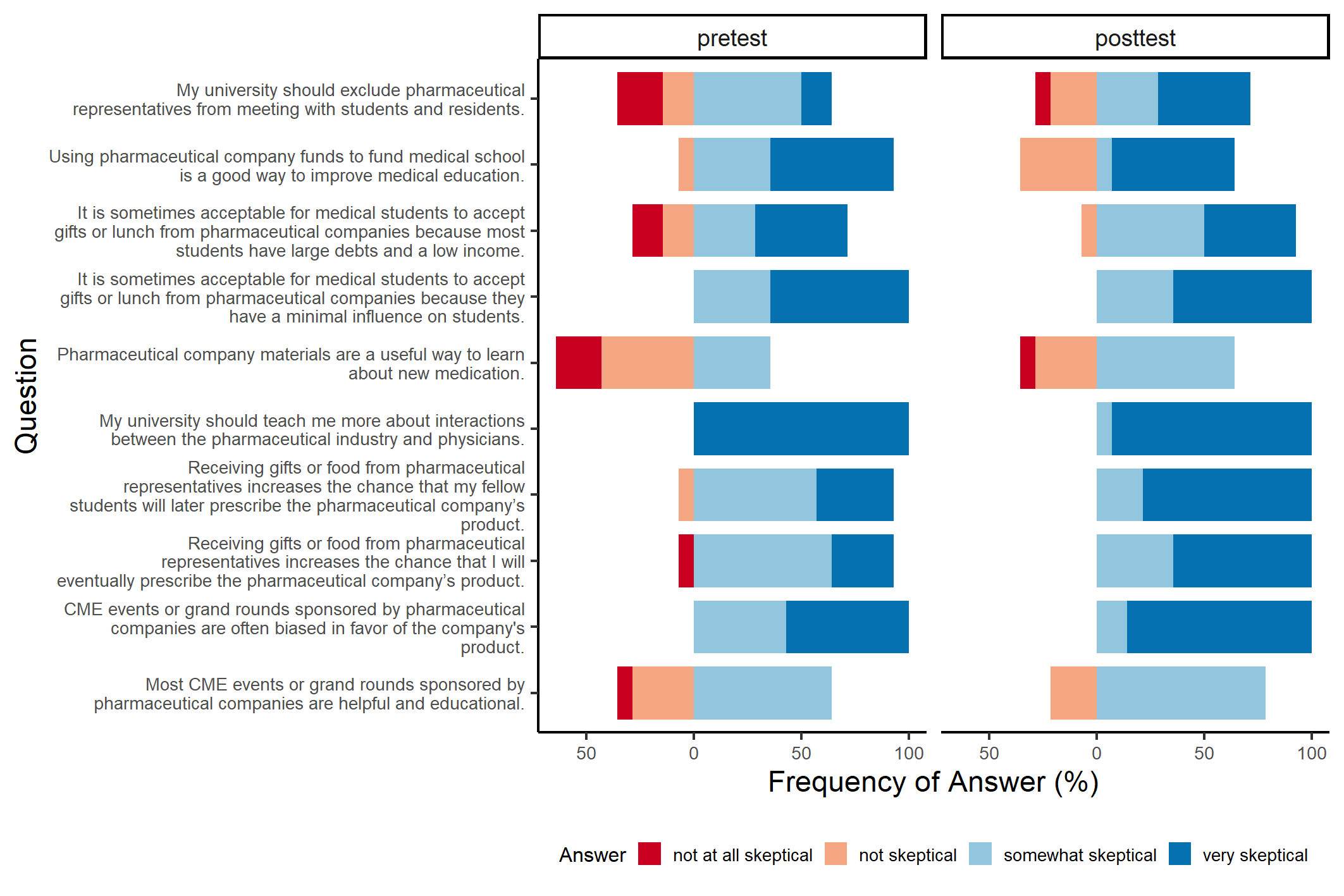

Supplement: Supplementary file 4 — Additional file 4: Figure S1. Individual item results of the attitude questionnaire [file 12909_2020_2332_MOESM4_ESM.png]
