# Supplementary material for: The feasibility and effectiveness of a blended-learning course for detecting and avoiding bias in medical data: a pilot study
Source: BMC Med Educ. 2020 Nov 7;20:408. doi: 10.1186/s12909-020-02332-w (PMC7648418; doi:10.1186/s12909-020-02332-w)
Supplement: Supplementary file 5 — Additional file 5: Table S2. Individual item data for the attitude questionnaire. [file 12909_2020_2332_MOESM5_ESM.docx]

Additional Table 2: Individual item data for the attitude questionnaire.

| **Item** | **Pre-test** | **Post-test** |
| --- | --- | --- |
| Most CME events or grand rounds sponsored by pharmaceutical companies are helpful and educational.* | 2.57 (0.65) | 2.79 (0.43) |
| CME events or grand rounds sponsored by pharmaceutical companies are often biased in favor of the company's product.* | 3.57 (0.51) | 3.86 (0.36) |
| Receiving gifts or food from pharmaceutical representatives increases the chance that I will eventually prescribe the pharmaceutical company’s product.* | 3.14 (0.77) | 3.64 (0.50) |
| Receiving gifts or food from pharmaceutical representatives increases the chance that my fellow students will later prescribe the pharmaceutical company’s product.* | 3.29 (0.61) | 3.79 (0.43) |
| My university should teach me more about interactions between the pharmaceutical industry and physicians. | 4.00 (0) | 3.93 (0.27) |
| Pharmaceutical company materials are a useful way to learn about new medication. | 2.14 (0.77) | 2.57 (0.65) |
| It is sometimes acceptable for medical students to accept gifts or lunch from pharmaceutical companies because they have a minimal influence on students. | 3.64 (0.50) | 3.64 (0.50) |
| It is sometimes acceptable for medical students to accept gifts or lunch from pharmaceutical companies because most students have large debts and a low income. | 3.00 (1.10) | 3.36 (0.63) |
| Using pharmaceutical company funds to fund medical school is a good way to improve medical education.* | 3.50 (0.65) | 3.21 (0.97) |
| My university should exclude pharmaceutical representatives from meeting with students and residents.* | 2.57 (1.02) | 3.07 (1.00) |
| **These items were used to calculate the skepticism score* | | |
